# Application of ribonucleoside vanadyl complex (RVC) for developing a multifunctional tissue preservative solution

**DOI:** 10.1371/journal.pone.0194393

**Published:** 2018-03-14

**Authors:** Tzong-Ming Shieh, Chi-Yuan Chen, Chuen Hsueh, Cheng-Chia Yu, Chin-Chuan Chen, Tong-Hong Wang

**Affiliations:** 1 Department of Dental Hygiene, College of Health Care, China Medical University, Taichung, Taiwan; 2 Research Center for Industry of Human Ecology, Chang Gung University of Science and Technology, Tao-Yuan, Taiwan; 3 Graduate Institute of Health Industry Technology, Chang Gung University of Science and Technology, Tao-Yuan, Taiwan; 4 Tissue Bank, Chang Gung Memorial Hospital, Linko, Taiwan; 5 Department of Anatomic Pathology, Chang Gung Memorial Hospital, Linko, Taiwan; 6 Institute of Oral Sciences, Chung Shan Medical University, Taichung, Taiwan; 7 Graduate Institute of Natural Products, Chang Gung University, Taoyuan, Taiwan; 8 Liver Research Center, Chang Gung Memorial Hospital, Linko, Taiwan; Johns Hopkins University, UNITED STATES

## Abstract

The quality of biological samples greatly affects the accuracy of scientific results. However, RNA in cryopreserved tissues gradually degrades during storage, leading to errors in the results of subsequent experiments. A suitable sample preservative solution can prolong storage and enhance the research value of samples. Here, we developed a sample preservative solution using the properties of the ribonucleoside vanadyl complex (RVC) and compared its effects on RNA and DNA quality, protein activity, and tissue morphology with the commercially available and widely used RNAlater® Stabilization Solution. The results showed that both the RVC-based preservative solution and RNAlater can effectively delay RNA degradation in tissue samples stored at 4°C or −80°C compared with samples stored without any preservative solution. In contrast to RNAlater, the RVC-based preservative solution did not result in damage to the tissue morphology or a loss of protein activity. Additionally, the RVC-based preservative solution did not affect the RNA and genomic DNA contents of the tissue samples or the results of subsequent experimental analyses. An RVC-based reagent can be used as a multifunctional yet relatively inexpensive tissue preservative solution to provide a comprehensive and cost-effective method for preserving samples for tissue banks.

## Introduction

Freshly frozen biological samples are important resources for clinical research, and the quality of samples greatly affects the accuracy of research results, especially in experiments such as gene microarray analyses that require extremely high RNA quality in samples[1–5]. The RNA in cryopreserved tissues continues to degrade during storage [5–7]. Numerous studies have shown that the RNA integrity number (RIN) of RNA extracted from samples stored at −80°C for more than 10 years is typically lower than 5, and thus, the RNA is no longer usable for most molecular biological experiments[7]. Hence, a method to delay RNA degradation during the storage process is needed for a reliable sample repository.

Several storage reagents are currently used to delay RNA degradation in biological samples during storage, such as 100% ethanol and the widely used RNAlater® Stabilization Solution (Thermo Fisher Scientific, Waltham, MA, USA)[8–14]. However, these stabilization solutions are associated with various drawbacks. For instance, 100% ethanol and RNAlater cause tissue dehydration, thus affecting cell morphology and causing protein denaturation in tissue samples[15, 16]. These problems restrict subsequent experimental analyses. There is currently no commercially available tissue preservative solution that can delay RNA degradation without affecting the tissue morphology and protein activity in tissue samples. Therefore, our study aimed to develop a multifunctional tissue preservative solution for sample storage to overcome these drawbacks.

The ribonucleoside vanadyl complex (RVC) was discovered in 1979, and studies have shown that it can inhibit the activity of various RNases[17–19]. Although its mechanism of action is not yet clear, RVC has often been used as an additive for the prevention of RNA degradation during RNA isolation[20, 21]. Recent studies have also revealed that RVC can inhibit the growth of *Staphylococcus aureus* and *Escherichia coli* by suppressing the formation of its ribosomal subunits and can serve as an antimicrobial additive[22, 23]. No reports have suggested that RVC can affect the protein activity in cells. Therefore, based on the above characteristics, we attempted to exploit the properties of RVC to develop a tissue preservative solution. Additionally, we performed a parallel assessment to compare the new solution to the commercially available RNAlater in terms of the maintenance of RNA and DNA quality, tissue morphology, and protein activity.

The results of our study revealed that, compared with RNAlater, the RVC-based preservative solution exhibited a more comprehensive protective effect on tissue morphology and on the quality of RNA, proteins, and genomic DNA. Although the RVC-based preservative solution was slightly inferior to RNAlater (RIN = 7.0 versus 7.3) in terms of delaying RNA degradation at 4°C, the new solution did not affect tissue morphology and protein activity in cells. Compared with RNAlater, which has some disadvantages including tissue dehydration and loss of protein activity, RVC may be a more useful multifunctional tissue preservative solution.

## Materials and methods

### Preparation of the preservative solution

The RNAlater used in this experiment was purchased from Thermo Fisher Scientific (Waltham, MA, USA), and RVC was purchased from Sigma-Aldrich (St. Louis, MO, USA). To determine the effects of different RVC concentrations on sample preservation, RVC was diluted with nuclease-free PBS containing a cryoprotectant (15% glycerol or 5% DMSO) to final concentrations of 0.2, 2, 20, and 40 mM. The resulting RVC-based preservative solutions were stored at −20°C until use.

### Mice

Six- to eight-week-old male BALB/c mice (purchased from the National Laboratory Animal Center, Taipei, Taiwan) were housed under pathogen-free conditions under a 12 h light/12 h dark cycle and fed autoclaved standard chow and water. The mice were bred at the animal center of Chang-Gung Hospital (Taoyuan, Taiwan), according to the Guidelines for the Care and Use of Laboratory Animals (NIH). All the animal experiments were approved by the Institutional Animal Care and Use Committee (IACUC) of Chang-Gung Hospital.

### Tissue processing

Liver tissue from 12-week-old male mice was used for testing. The mice were sacrificed with an anesthetic overdose of xylazine 5 mg/kg plus ketamine 30 mg/kg, and the liver tissue was excised from the mice and evenly sliced into small pieces (2 × 2 cm) using a scalpel. Each tissue sample was placed in a cryogenic vial containing 300 μL of a preservative solution so that the preservative solution covered the entire tissue sample, and the vials were stored at 4°C for 7 days or at −80°C for 1 month prior to RNA and DNA isolation and quality assessment.

All experiments related to the animal studies were approved by the Institutional Animal Care and Use Committee (IACUC) at Chang-Gung Hospital, and the methods were carried out in accordance with the guidelines for the Care and Use of Laboratory Animals (NIH).

### RNA and genomic DNA isolation and quality assessment

Mouse tissue samples stored in different preservative solutions were subjected to the isolation of RNA and genomic DNA using an RNeasy Mini Kit (QIAGEN, Hilden, Germany) and a QIAmp DNA Extraction Kit (QIAGEN), respectively. To minimize artificial error, both DNA and RNA isolation were performed using a QIAcube automated isolation workstation (QIAGEN). A fragment of the *IFN-r* gene was amplified from the extracted DNA by PCR using 2X Taq PCR MasterMix (BIOTOOLS CO., LTD., Taiwan), and the purified PCR products were subjected to Sanger sequencing to evaluate the integrity of the genomic DNA. The RIN was calculated using the 18S and 28S ribosomal RNA peak fractions, which were obtained by capillary electrophoresis and served as standards to assess the RNA quality in freshly frozen tissue samples using an Agilent 2100 Bioanalyzer (Agilent Technologies, Santa Clara, CA, USA). In addition, we analyzed the expression of twelve microRNAs, namely miR-96, miR-135b, miR-139-5p, miR-147b-3p, miR-150-5p, miR-183, miR-184-3p, miR-186-5p, miR-190a-5p, miR-195-5p, miR-200c-3p, and miR-338-3p, by quantitative real-time reverse transcription (RT)-PCR using *snoRNA202* as an internal control to evaluate the protective effect of the preservative solutions on microRNAs.

### Whole transcriptome library preparation and sequencing

All procedures were carried out according to the manufacturer’s protocol (Illumina Inc., San Diego, CA). The SureSelect Strand Specific RNA Library Preparation Kit (Agilent Technologies) was used for 75SE (Single-End or Paired-End) sequencing of all the samples on the Solexa platform for construction of the library. The sequence was directly determined by the sequencing-by-synthesis technology using the TruSeq SBS Kit (Illumina). Raw sequences were obtained from the Illumina Pipeline software bcl2fastq v2.0, which was expected to generate 10 million reads per sample.

### RNA-seq analysis

The generated sequences were filtered to obtain good quality reads. Trimmomatics was implemented to trim or remove the reads according to the quality score. High-quality reads obtained after filtering the low-quality data were analyzed using TopHat/Cufflinks[24] for the estimation of gene expression. The level of gene expression was quantified as FPKM (fragments per kilobase of transcript per million mapped reads) values. For the differential expression analysis, CummeRbund was employed to perform statistical analyses of the gene expression profiles. The reference genome and gene annotations were retrieved from the Ensembl database.

### Analysis of cellular morphology

In addition to directly examining the physical appearance of tissues, mouse tissue samples (processed with different preservative solutions and under different storage conditions) were fixed with 10% formaldehyde, dehydrated, and embedded in paraffin. The paraffin-embedded tissue samples were sectioned and subjected to hematoxylin and eosin (H&E) staining; the cellular morphological features were examined by two pathologists.

### Immunohistochemical staining

Mouse tissue samples stored in different preservative solutions were fixed with 10% formaldehyde and embedded in paraffin. Next, the paraffin-embedded tissue samples were sliced using a microtome (2-μm-thick slices) for immunohistochemical staining. Briefly, the paraffin-embedded tissue samples were baked at 70°C in an oven for 1 hour and then dewaxed with xylene followed by rehydration with 100%, 95%, 75%, and 50% ethanol. Thereafter, a BOND-MAX Automated IHC/ISH Stainer (Leica Microsystems GmbH, Wetzlar, Germany) was used for staining, and the expression and distribution of EGFR and PCNA proteins in the tissue samples were assessed by two pathologists.

### Analysis of protein activity

Protein activity was analyzed by assessing luciferase activity and light emission by green fluorescent protein (GFP). The human squamous cell carcinoma cell line (SAS), which stably expresses the luciferase reporter plasmid, was used to analyze luciferase activity. Briefly, the cells were harvested using GLO-lysis buffer (Promega Corporation, Madison, WI, USA), and 60 μL of the cell lysate was thoroughly mixed with 10 μL of various preservative solutions. After incubation at 4°C for different times, the effects of different preservative solutions on luciferase activity were measured using a Fluoroskan Ascent™ FL Microplate Fluorometer and Luminometer (Thermo Fisher Scientific).

GFP activity was determined via direct examination under a microscope. First, the culture medium was aspirated, and the cells were washed twice with PBS. Then, 1 mL preservative solution was added to the SAS cells that stably expressed GFP. After 10 minutes of cultivation at 25°C, the intensity of GFP fluorescence was examined, and the cells were photographed using a fluorescence microscope.

### Statistical analysis

The original real-time PCR data and data of the luciferase activity assay were recorded as continuous variables and analyzed using Student’s *t*-test. All statistical analyses were performed using SPSS 16.0 and Excel 2007. All statistical tests were two-sided, and p-values < 0.05 (*), < 0.01 (**), or < 0.001 (***) were considered to be significant.

## Results

### Both the RVC-based preservative solution and RNAlater can effectively delay RNA degradation

**Fig 1:**
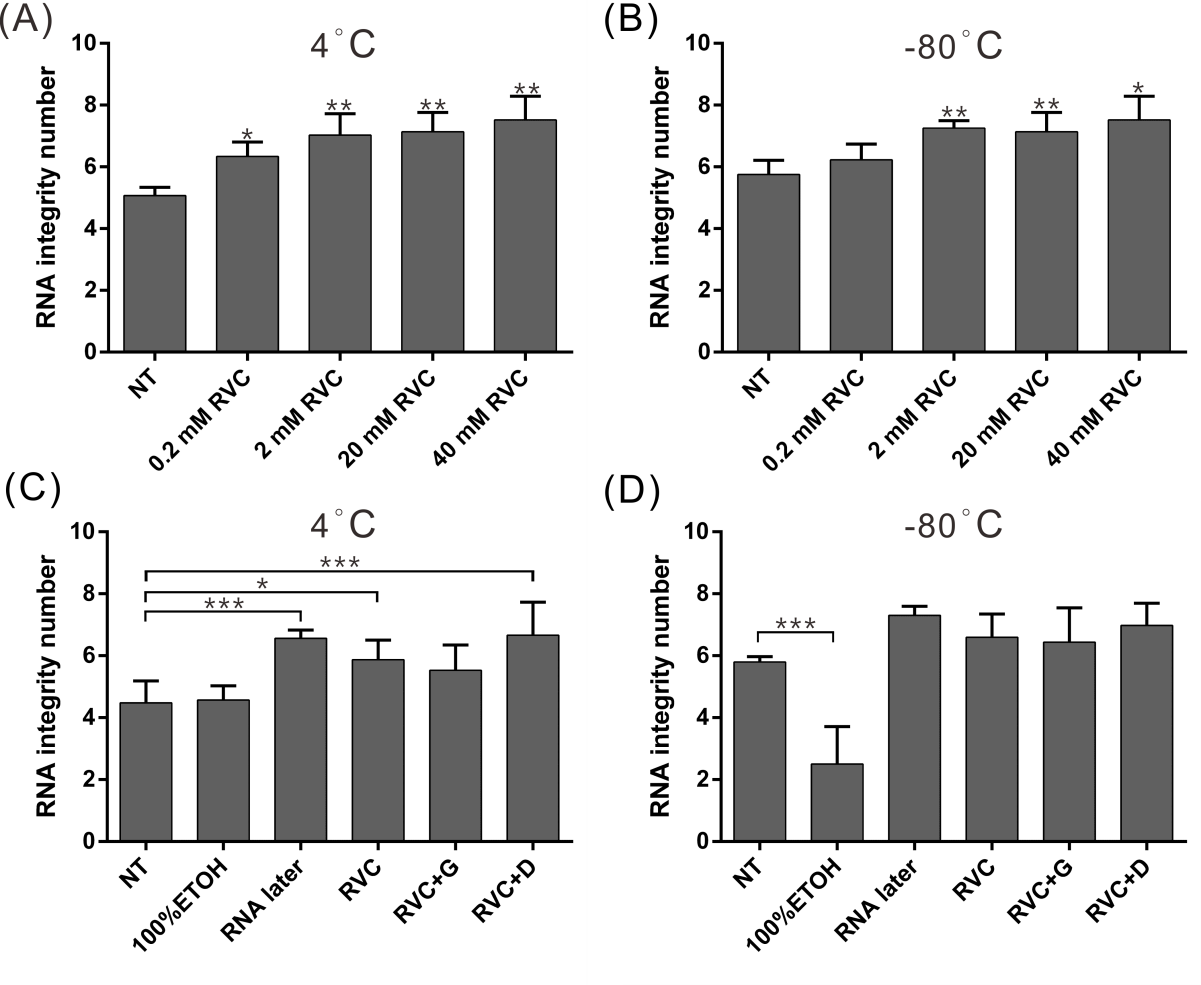
Both the RVC-based preservative solution and RNAlater can effectively delay RNA degradation. (A-B) Quality of RNA in tissue samples (n = 3) preserved with different concentrations of RVC in the preservative solution at 4°C or −80°C for 7 days. (C) Quality of RNA in tissue samples (n = 5) preserved in different preservative solutions at 4°C for 7 days and −80°C for one month (D). NT: stored without any preservative solution; RVC+G: RVC+15% glycerol; RVC+D: RVC+5% DMSO; * p < 0.05, ** p < 0.01, *** p < 0.001 (compared to the NT group).

To optimize the concentration of RVC in the preservative solution, we evenly divided the fresh liver tissue of mice and stored samples in preservative solutions with different concentrations of RVC, including 0.2, 2, 20, and 40 mM, at 4°C or −80°C for 7 days prior to RNA isolation and quality assessment (Fig 1A and 1B). We found that 2, 20, and 40 mM RVC-based preservative solutions exhibited the best preservative effects on the tissue RNA, which were significantly better than the effect of 0.2 mM RVC and the control condition (tissue samples stored without immersion in any preservative solution). Nonetheless, no significant difference was observed between 2 and 40 mM RVC-based preservative solutions, indicating that RVC concentrations higher than 2 mM have the best preservative effects. Hence, the 2 mM RVC-based preservative solution (RVC alone and RVC in combination with DMSO or glycerol) was used for subsequent experiments.

To compare the protective effects on tissue RNA between the RVC-based solution and RNAlater, we evenly divided the fresh liver tissue samples of mice into groups for storage in different preservative solutions. The tissue samples were stored at 4°C for 7 days or at −80°C for 1 month prior to RNA isolation and quality assessment to compare the tissue-preservative effects of the preservative solutions under different conditions (Fig 1C and 1D). According to the results, the preservative effects of both RVC and RNAlater on the samples stored at 4°C were better than the effects of 100% ethanol and the no-treatment control. The mean RIN values of the RNAlater group and the RVC-based preservative solution group were 6.56 and 6.04, respectively, whereas the RIN values of samples stored at 100% ethanol and the no-treatment control were 4.48 and 4.58, respectively (Fig 1C). The results indicated that both RVC and RNAlater can effectively delay RNA degradation in tissue samples. At −80°C, the RIN values of the tissue samples stored in either RNAlater or the RVC-based preservative solution were greater than 6.5 and were better than the RIN values of tissue samples stored without any preservative solution (RIN value of 5.8; Fig 1D), indicating that both the RVC-based solution and RNAlater have a favorable preservative effect on RNA at either 4°C or −80°C.

### RVC-based preservation solutions have minimal effects on gene expression when compared to fresh-frozen tissue

**Fig 2:**
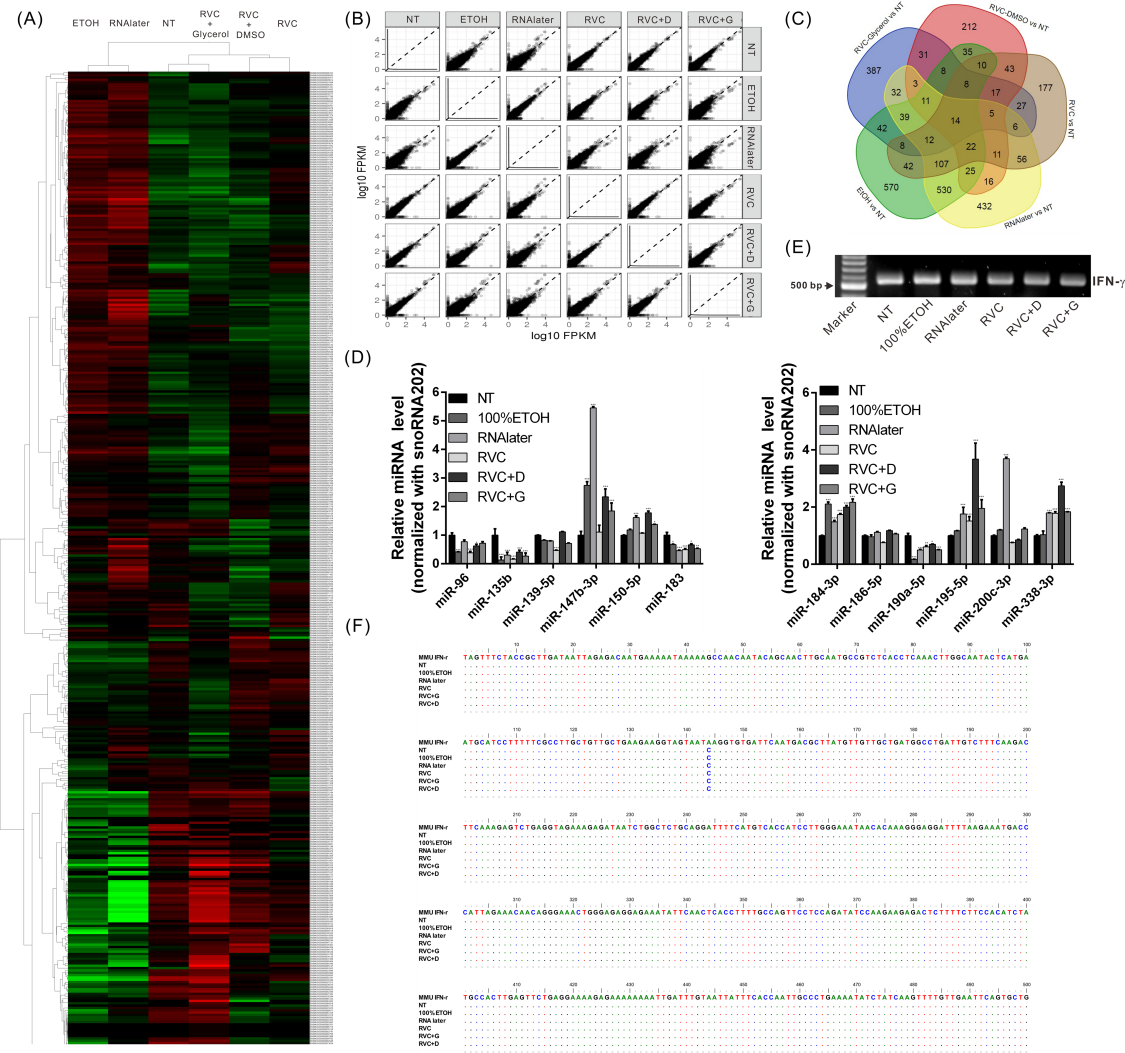
Different types of preservative solutions cause changes in the microenvironment of cells and affect gene expression levels. (A, B) RNA was isolated from tissue samples immersed in different preservative solutions at −80°C for 1 month and subjected to whole-transcriptome analysis. Different preservative solutions affected the gene expression levels within tissue samples to different degrees. The total number of genes affected by the different preservation solutions compared to the control group are shown in (C). (D) The RNA from the abovementioned samples was analyzed for the expression of 12 miRNAs by real-time RT-PCR using *snoRNA202* as an internal control. Different preservative solutions also affected the expression of miRNA in the tissue samples. * p < 0.05, ** p < 0.01, *** p < 0.001 (compared to the NT group). (E) The genomic DNA was isolated from the samples processed as described above and subjected to PCR for amplification of an *IFN-r* gene fragment. (F) The PCR-amplified *IFN-r* gene fragments were subjected to sequence analysis by the Sanger sequencing method. The correct sequences were obtained from the samples stored using all preservation methods.

To determine whether different preservative solutions affect the expression of genes in the specimen, mouse liver tissue samples were immersed and stored in different preservation solutions at −80°C. After one month, cellular RNA was extracted and comprehensively analyzed to detect differences in the gene expression profiles between groups using whole-transcriptome analysis. As shown in Fig 2A and 2B, the five preservation solutions affected the gene expression levels in the tissue samples to different degrees. Compared with the control group that was not immersed in any preservation solution, RNAlater and 100% ethanol had the greatest effects on gene expression, whereas the RVC-based preservation solutions had relatively minimal effects on gene expression. Fig 2C shows the total number of genes affected by the different preservation solutions compared to the control group that was not immersed in any preservation solution. There were 432 genes affected by RNAlater and 570 genes affected by 100% ethanol. However, significantly fewer genes were influenced by the RVC-based preservation solutions than by the RNAlater and ethanol solutions (RVC: 177 genes, RVC+DMSO: 212 genes, RVC+Glycerol: 387 genes). These results indicated that after infiltrating the tissue, different preservative solutions may cause various changes in the microenvironment of tissues, thereby affecting the expression of genes within the preserved tissues.

In addition to the mRNA molecules, microRNAs are also commonly evaluated in biological investigations. Hence, the effects of the aforementioned preservation solutions on the expression of 12 microRNAs (miRNAs) were also analyzed by real-time RT-PCR using *snoRNA202* as an internal control. The experimental results showed that all miRNAs could be successfully amplified from the specimens stored in the different preservative solutions. However, similar to the results of the whole-transcriptome analysis, the five preservation solutions affected the miRNA expression levels to different extents. In addition, the different solutions induced different regulatory trends for the various miRNAs evaluated (Fig 2D). These results demonstrated that different preservative solutions also affect the expression of miRNA in tissues.

### Neither RVC nor RNAlater affect the genomic-DNA content or results of subsequent experiments

Numerous clinical studies, such as point mutation assays or promoter methylation assays, require the analysis of genomic DNA sequences in a biological sample. To determine whether the preservative solutions affect the quality of genomic DNA, we isolated genomic DNA from samples stored in different preservative solutions, followed by PCR amplification of different regions of the IFN-*r* gene (Fig 2E). The results showed that the expected amplification product could be successfully obtained from samples stored in RNAlater or RVC-based preservative solutions. We next performed sequence analysis of the purified PCR products and found that the DNA sequences were consistent with those of the control group samples, which were stored without any preservative solution (Fig 2F). These findings indicate that none of the preservative solutions affected the DNA structure or components in the tissue samples or the results of subsequent experiments.

### The RVC-based preservative solution only causes minor negative effects on intracellular protein activities

**Fig 3:**
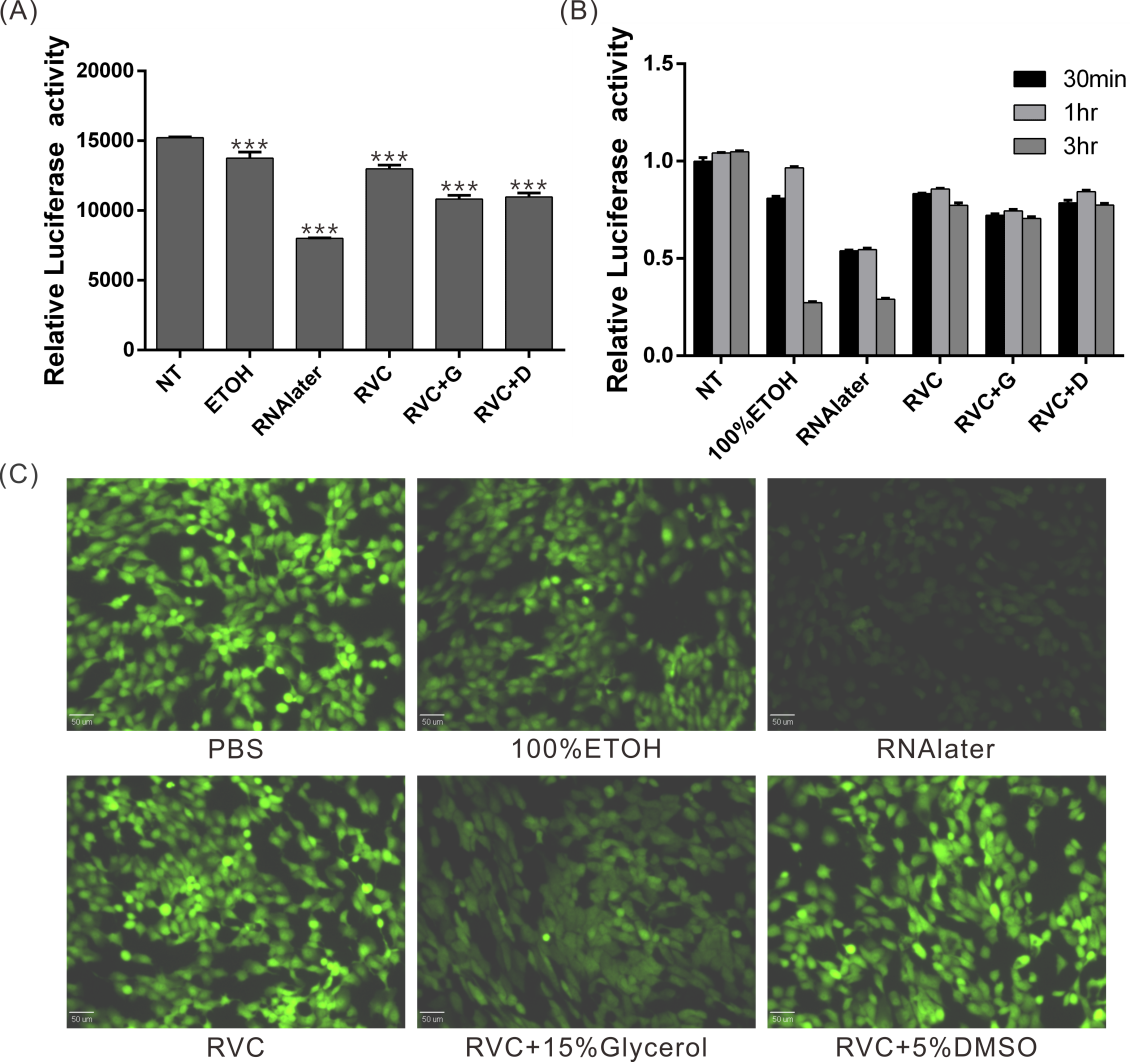
The RVC-based preservative solution has a milder effect on protein activity than 100% ethanol and RNAlater. (A-B) Determination of the effect of different preservative solutions on intracellular luciferase activity. The lysate of cells expressing luciferase was reacted with different preservative solutions, and luciferase activity was measured after 0, 0.5, 1, and 3 hours (n = 3). *** p < 0.001 compared to the NT group. (C) The effects of various preservative solutions on intracellular GFP activity. The RVC-based preservative solution exerted a significantly milder effect on GFP fluorescent activity than 100% ethanol and RNAlater.

Many studies require the determination of protein activity. To elucidate the effects of our preservative solution on protein activity, we harvested SAS cells stably expressing luciferase and treated the cell lysates with different preservative solutions. Luciferase activity was then measured at 0, 0.5, 1, and 3 hours after the reaction (Fig 3A and 3B). The results revealed that luciferase activity was reduced by 45% compared with that in the control group after 0.5 hours of treatment with RNAlater and was reduced by ~72% after 3 hours of treatment. In contrast, RVC and RVC + 5% DMSO induced milder effects on luciferase activity, even after 3 hours of treatment (~25% reduced activity, Fig 3B). These results showed that the RVC-based preservative solution has a significantly milder effect on protein activity than RNAlater and 100% ethanol solutions. Additionally, we evaluated the effects of the preservative solutions on intracellular GFP activity using fluorescence microscopy (Fig 3C) and obtained similar results. Thus, RVC and RVC + 5% DMSO have reduced effects on protein activity compared with 100% ethanol and RNAlater, which induce a significant loss of GFP fluorescence.

### The RVC-based preservative solution does not affect tissue morphology

**Fig 4:**
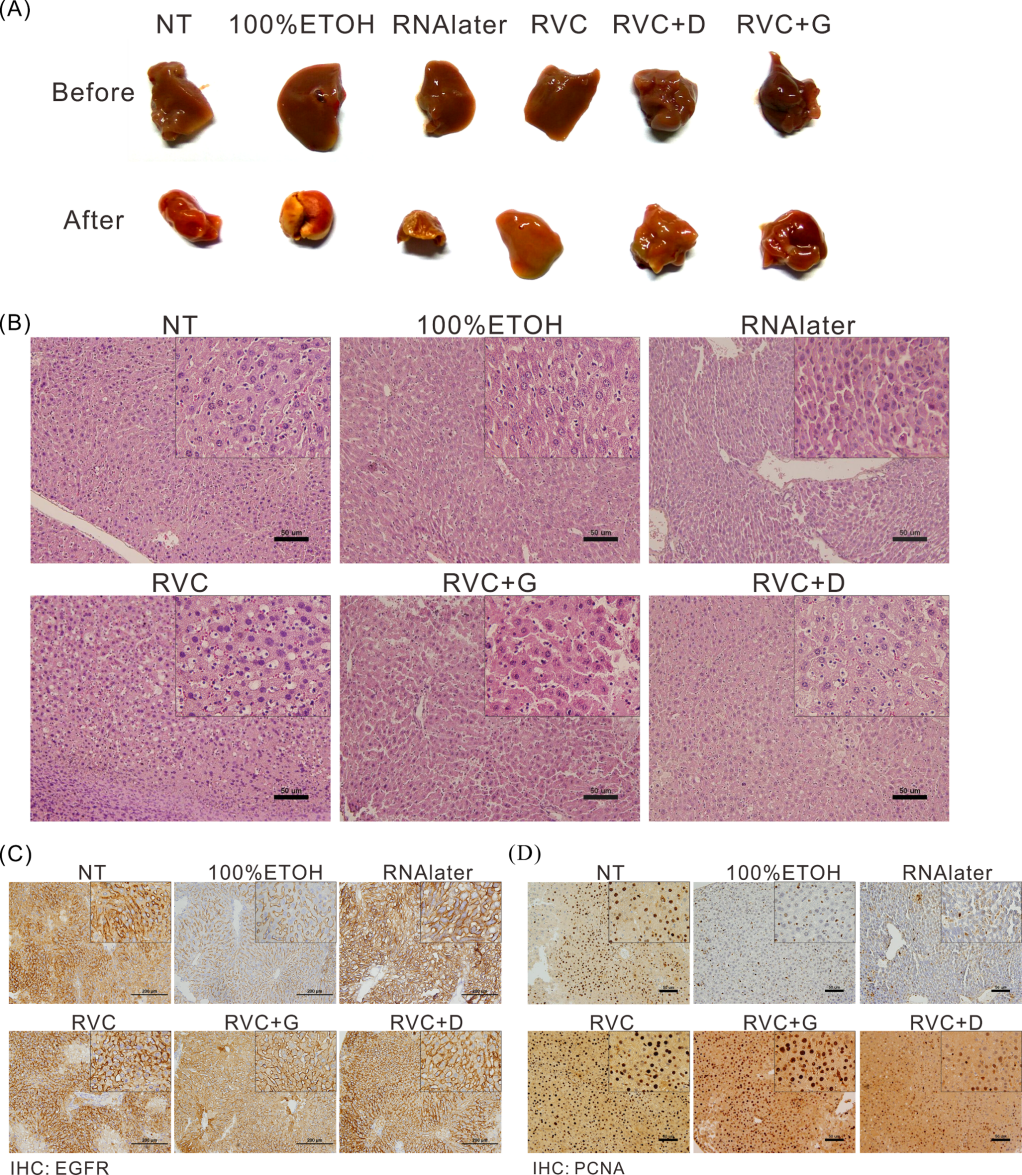
RVC does not affect tissue morphology or the expression and distribution of proteins in tissue samples. (A) Morphological features of murine liver tissue samples stored in different preservative solutions. (B) The abovementioned paraffin-embedded tissue samples were sliced and stained with H&E for the examination of cellular morphological characteristics. RNAlater and 100% ethanol caused significant tissue dehydration, whereas the RVC-based preservative solution did not cause this phenomenon. (C-D) Determination of EGFR and PCNA protein expression and distribution by immunohistochemical staining in tissue samples treated with different preservative solutions. The results from the RVC-based groups were consistent with the data of the control group.

After storage for a certain amount of time, the cell morphology of many types of tissues must be re-validated to ensure that the tissue has retained its original properties. Hence, a good preservative solution should not affect tissue morphology to ensure that the results of subsequent clinical interpretations are valid. To determine whether these preservative solutions affect cell morphology, we examined the physical appearance of tissue samples stored in different preservative solutions (Fig 4A). Additionally, the tissue samples were embedded in paraffin, sliced, and subjected to H&E staining (Fig 4B). We found that 100% ethanol and RNAlater caused significant tissue dehydration compared with the control group, whereas the RVC-based preservative solution did not cause this phenomenon (Fig 4A and 4B). The results confirmed that RVC does not significantly alter the morphological features of tissue, in contrast to RNAlater and 100% ethanol. In addition, we determined the expression of EGFR (transmembrane protein) and PCNA (nuclear protein) proteins by using immunohistochemical staining, and found that the staining results and protein distributions in the RVC-based preservation groups were consistent with the data in the control group (Fig 4C and 4D). The results indicate that RVC-based preservative solutions do not affect certain types of subsequent procedures, such as immunohistochemical staining.

## Discussion

In clinical research, sample quality can greatly affect the results of a study. For example, high-quality DNA and RNA are required for studies that include analyses of gene mutation sites, biomarker screening, or gene expression regulation, and a good sample preservative solution can ensure that researchers acquire accurate results. In this study, we exploited the properties of RVC to develop an effective tissue preservative solution that does not affect tissue morphology or protein activity while conferring a preservative effect (on tissue RNA and DNA) similar to that of the widely used RNAlater reagent. The new reagent acts as a multifunctional tissue preservative solution.

The RVC compound was discovered in 1979 and has been widely used as an additive in RNA isolation or staining reagents to delay RNA damage in tissues caused by the dye during staining[25–27]. Nonetheless, no reports have comprehensively assessed the effects of RVC on the quality of DNA, protein activities, and tissue morphology. To the best of our knowledge, this study is the first to investigate the effects of RVC on the abovementioned parameters and to assess the suitability of RVC for use in a tissue preservative solution. In this study, we found that the RVC-based preservative solution causes less tissue damage than the more widely used RNAlater® Stabilization Solution. In addition, the proposed reagent is cheaper than RNAlater and, thus, can effectively reduce the operating costs of studies incorporating a large sample library (Table 1).

**Table 1:** Comparison of the effects of five preservatives on tissue storage.

	100% ETOH	RNAlater	RVC only	RVC+5% DMSO	RVC+15% Glycerol
Preservation of DNA	+++	+++	+++	+++	+++
Preservation of RNA	+	+++	+++	+++	+++
Maintenance of protein activity	+	−	+++	+++	++
Maintenance of tissue morphology	−	−	+++	+++	+++
Influence on gene expression	higher	higher	lower	lower	moderate
Cost/unit (USD)	$0.002	$0.270	$0.045	$0.053	$0.048

+++: Excellent

++: Satisfactory

+: Poor

−: Failure

In the experiment, we found that different preservative solutions affect the results of gene expression analysis to varying degrees. In particular, 100% ethanol had the most significant effect, indicating that after infiltrating the tissue, preservative solutions may cause changes in the microenvironment of cells, thereby affecting the gene expression. Hence, cells or tissues subjected to relevant experiments should be kept in the same preservative solution to eliminate its effects on the gene expression. Moreover, the results shown in Fig 1C and 1D demonstrate that RNAlater can achieve a slightly better preservative effect than RVC only with respect to RNA quality. Thus, RNAlater may be considered as an optimal preservative for research in which RNA quality is an essential requirement (such as for RNAseq and microarray analyses) or if only an RNA-related analysis is required. On the other hand, the protein activity analysis revealed that the activity of luciferase decreased to ~30% after treatment with RNAlater or 100% ethanol for 3 hours, indicating that RNAlater and 100% ethanol may denature the luciferase protein and inhibit its activity. In contrast, the RVC-based preservative solution had almost no effect on luciferase activity, indicating that RVC does not affect protein structure and stability. We also confirmed the inhibitory effect of RNAlater and 100% ethanol on the activity of green fluorescent protein (GFP). Nevertheless, the results showed that the RVC+Glycerol preservative solution reduces the fluorescence intensity of GFP, possibly because of the addition of glycerol. Similarly, previous relevant reports have noted that glycerol and sucrose shorten the lifetime of GFP [28, 29]. The above assays indicated that the use of RVC+5% DMSO as a tissue preservative solution exerts the most comprehensive preservative effect on DNA, RNA, and protein.

Previous studies have shown that in addition to inhibiting the activities of many ribonucleolytic enzymes such as RNase, a high concentration of RVC (10 mM) can inactivate reverse transcriptase and many restriction endonucleases or interfere with subsequent experiments such as reverse transcription (RT) and PCR by competing with dNTPs [19, 30–32]. However, the RVC preservation solution did not influence the RT-PCR results in our experiments. We infer that 2 mM RVC preservation solution does not affect the activities of the aforementioned proteins. Nevertheless, as shown in Fig 3B, the activity of luciferase was slightly inhibited by RVC, indicating that RVC still inhibits the activities of certain proteins. Therefore, it is recommended to determine the existence of any crosstalk between proteins and RVC in experiments aimed to analyze protein activities of proteins purified from RVC-preserved tissues. This should be evaluated prior to the analysis to determine if the RVC preservation solution is applicable for tissue preservation. In addition, RVC was reported to rapidly oxidize following exposure to oxygen in the air, which reduces its inhibitory effect on RNase[33]. This might be the reason for the inferior preservative effect of the RVC preservation solution on the tissue RNA detected at 4°C compared to that observed at −80°C (Fig 1C). Hence, it is recommended to store tissue samples at −80°C to achieve an optimal preservative effect if RVC preservation solution is used for long-term tissue preservation.

DNA, proteins, and tissue morphology analyses and RNA assays are very important for molecular and cell biology studies. A good tissue preservative solution must not affect the abovementioned intracellular components (such as DNA, RNA, and proteins) or tissue morphology while maintaining RNA quality. Although our study is based on only a 1-month observation, we found that the RVC-based preservative solution has a preservative effect (on RNA) similar to that of RNAlater and exerts a good preservative effect on microRNA. Additionally, the analysis of luciferase and GFP activities revealed that the RVC-only solution and the RVC + 5% DMSO solution have significantly milder effects on protein activity than RNAlater. However, RVC + 15% glycerol reduces GFP activity, and the detailed mechanism requires further investigation. Although we did not assess longer durations, we demonstrated multiple useful functions of the RVC-based preservative solution, including that the quality of DNA, mRNA, miRNA, and proteins in tissue samples can be preserved over a longer period in samples treated with RVC solution compared to control samples. In future studies, we will determine whether RVC can preserve other types of samples, such as blood plasma or blood cells. In summary, the results of this study reveal a cheaper and multifunctional method for preserving tissue samples. For studies that require good preservation of tissue morphology, protein activity and RNA quality, the RVC-based preservative solution is a good alternative for tissue preservation. Furthermore, for a large-scale prospective tissue collection (such as a biobank), the use of RVC-based preservation solutions could not only maintain the quality and characteristics of the specimens, but also effectively reduce the cost of preservation.
